# What Are the Benefits and Risks of Fitting Patients with Radiofrequency Identification Devices

**DOI:** 10.1371/journal.pmed.0040322

**Published:** 2007-11-27

**Authors:** Mark Levine, Ben Adida, Kenneth Mandl, Isaac Kohane, John Halamka

## Abstract

Background to the debate: In 2004, the United States Food and Drug Administration approved a radiofrequency identification (RFID) device that is implanted under the skin of the upper arm of patients and that stores the patient's medical identifier. When a scanner is passed over the device, the identifier is displayed on the screen of an RFID reader. An authorized health professional can then use the identifier to access the patient's clinical information, which is stored in a separate, secure database. Such RFID devices may have many medical benefits—such as expediting identification of patients and retrieval of their medical records. But critics of the technology have raised several concerns, including the risk of the patient's identifying information being used for nonmedical purposes.

## Abstract 2

In 2004, the FDA approved an RFID device that is implanted under the skin of the upper arm of patients and that stores the patient's medical identifier.

## Mark Levine's Viewpoint: RFID Devices Have the Potential to Improve Medical Care

Radiofrequency identification devices are tiny, potentially implantable appliances that can store clinical information that is able to be captured remotely. Their use has the potential to make significant advances in the effectiveness, efficiency, and safety of medical care by improving patient identification, promoting patient safety, and expediting access to patients' medical records. Yet, as with all new technologies, their adoption must be tempered by attention to potential unintended consequences.

Today's implantable RFID devices are passive instruments capable of short-range transmission only when activated by an external energy source, such as a radio transmitter. The United States Food and Drug Administration (FDA), which regulates medical devices, has not yet approved self-powered, or active, devices. The information stored on a passive RFID appliance cannot be edited or changed. It may be accessed by exposing the device to a predetermined radiofrequency at a sufficiently close range. The device converts this external energy into a signal that can be received and translated by the transmitter. The information thus captured is specific to the person carrying the implanted appliance or to the device to which it is attached (such as a surgical sponge).

Ethical concerns regarding the use of RFID devices arise from issues pertaining to informed consent, the privacy and accessibility of stored information, and the purposes for which the transmitted data will be used. Patients must trust that RFID devices will not be implanted or removed without their prior consent. When seeking patients' consent to implant an RFID device, physicians must do two things. First, they must disclose the possibility of unauthorized access to the information stored on the device. Second, they must allow patients to determine how their stored information is to be used, and who will have access to it.

Patients must also be confident that their personal information will be used solely for clinically beneficial purposes. Physicians must therefore take additional responsibility for ensuring that human-implantable RFID devices are used only to improve patient care and are not abused for nonclinical ends, such as identification of the presence, age, and/or other personal information of an individual. Moreover, physicians must take efforts to ensure that implanted devices are able to keep clinical data confidential and protected from unauthorized access. Such unauthorized access could potentially result in social discrimination, the loss of health care coverage, or the publication of potentially sensitive medical information.

Physicians do not bear sole responsibility for ensuring the safety of RFID devices. The FDA assists in protecting patients' confidentiality by requiring that patient-specific information contained in RFID devices consist only of a unique identifier that can be used to access patients' clinical records, which are stored in a separate, secure database. This two-step process of linking identification to an external data source greatly diminishes the likelihood that sensitive patient information will be disclosed to an unauthorized source.

The functional capabilities of RFID devices may continue to expand, especially if active RFID devices are approved by the FDA. It might then be possible for such devices to disclose the location of the wearer or to carry more explicit individual information that could be abused.

If the above concerns can be properly mitigated and if continued use of these devices confirms their potential to improve the quality of patient care, physicians would have an ethical obligation to advocate for their widespread adoption. At the same time, we should continue to examine the safety, efficacy, and social consequences of these devices as part of our constant commitment to improve patient care.

## Ben Adida, Kenneth Mandl, and Isaac Kohane's Viewpoint: RFID Implantation May Invade Privacy

The American Medical Association (AMA) recently issued a report on “Radio Frequency ID Devices in Humans,” which concluded that these small implantable devices “may help to identify patients, thereby improving the safety and efficiency of patient care” [1]. The AMA recommends that during the informed consent process for RFID implantation, patients should be told of “medical uncertainties associated with these devices.” However, health policy makers, doctors, and the public must understand that RFID devices, unlike other forms of medical technology, have an impact upon patients' privacy that extends far beyond the medical arena. With an implanted RFID device, individuals can be tracked surreptitiously by anyone using a generic RFID reader, available for just a few hundred dollars. The informed consent process needs to present this risk clearly, and the AMA should amend its report to specifically address this unusual risk.

An RFID chip is typically a simple piece of hardware with a unique identifier and a small amount of read/write storage. Currently, this storage is insufficient for significant medical information, so the chip usually stores only a patient identifier, which links to a complete electronic record stored separately. The AMA correctly warns that if patient data were eventually to be stored on these RFID devices, they should be protected with the same level of access control as that required of current medical record systems, using, for example, data encryption. What the current policy fails to address is that every RFID device publicly advertises its identifier. Even if the *patient* identifier were encrypted in the device's read/write storage, the *unique* identifier remains readable by any RFID reader—medical or nonmedical. In addition, RFID readers can function surreptitiously, at a distance of up to a few feet.

Consequently, RFID devices have been aptly described as “a kind of license plate for people” [2]. If such devices become widely deployed, they may provide an incentive for both well and ill-intentioned parties to set up readers for these “license plates.” A store owner might set up a reader to track frequent customers, linking the unique identifier to the customer record upon first purchase. Law enforcement might leverage RFID as a means of ubiquitous surveillance. Because the RFID identifier is of no medical significance, it is not protected by the Health Insurance Portability and Accountability Act (HIPAA), and there are no laws that regulate how and by whom it can be read; the possibilities for privacy invasion by inter-database linkage are vast.

A case study from another industry where RFID devices were implemented is informative. New US passports include an RFID chip that stores basic information about the passport holder. However, in response to criticisms of potential identity theft and privacy violation by surreptitious readings [3], three safeguards were put in place: (1) data on the passport are encrypted; (2) the encryption key is printed on the inside of the passport, so that physical access to the passport is required for decryption; and (3) the passport is shielded, so that even its unique RFID identifier cannot be read while closed.

It does not appear, from the AMA statement, that any of these safeguards have been considered for the medical use of RFID chips, and when the device is implanted, the latter two become somewhat difficult to implement. Until RFID devices have the capacity to store reasonable amounts of live medical data, a safer, less invasive, and less expensive approach may be a simple medical bracelet with the patient identifier printed as a barcode. In any case, the issue of surreptitious reads of the RFID identifier must be considered.

As personalized medicine incorporates a wider range of advanced technologies, these sorts of crossover consequences will become more frequent and we will need to heed lessons learned in nonmedical fields. Given the importance of privacy in health care, the AMA should set a strong privacy-friendly precedent with its RFID recommendation. There are many applications of RFID technology that can improve health care, but the implantation of these devices into patients merits a healthy dose of skepticism. At the very least, the informed consent process must transparently convey the significant societal side effects of RFID devices.

## John Halamka's Viewpoint: RFID Devices Enable Patients to Be Stewards of Their Own Health Data

In December of 2004, I was implanted with a VeriChip RFID device.

As a physician and chief information officer, I felt qualified to evaluate the medical, legal, moral, and privacy aspects of the device. After using the device for three years, I am not an evangelist for implanted RFID, but I believe it can be valuable for some patients who understand the risks and benefits.

My implantation process was simple—a five minute office procedure, which included disinfection of the implant site on my upper right arm, a few cubic centimeters of lidocaine, and insertion of the injector into my subcutaneous fascia. I did not experience pain, bleeding, or any post-procedure infection. The implant is not palpable, does not migrate, and has no physical side effects such as itching, irritation, or changes in skin appearance. The RFID device does not impede my activities; even while rock or ice climbing I have hit the implant site many times without any problems. The device is undetectable by airport security metal detectors and hand scanners.

One possible side effect is that my RFID device can be scanned by retail security systems using 134.2 kHz RFID technology, the frequency of my implant. I have had experiences at Home Depot and Best Buy where my device seemed to set off the anti-theft systems. My personal data are not readable by such systems, but they may be able to detect the presence of an implanted RFID tag.

Given my experience, what are the risks and benefits? The medical risks of any implant are infection, pain, keloid formation at the puncture site, and reaction to the local anesthetic. There are quite a range of nonmedical risks. After my implant, I received many e-mails saying that I had become a “Borg” and had lost some of my humanity because I was now a hybrid human/machine. Some e-mails even referred to the Book of Revelation, noting that I now carried the number of the Beast. Thus, chip carriers have a risk of being social outcasts.

The chip holds a static and unencrypted 16 digit number, which is used to point to a Web site containing personal health record data. The Web site requires a username and password, ensuring appropriate security. It is conceivable that a person on a subway could scan a patient's number without their knowledge and steal their medical identity by creating an identical chip and implanting it. This is a very theoretical risk because hospitals are not widely using implanted RFID chips as a means of identification. If the implanted chip were used for security purposes, such as opening a door to a secure area, the person who scanned the patient on the subway could replay the RFID signal and gain access to the secure area. Again, this is purely theoretical since implanted RFID devices are not often used as security authenticators.

If these are the potential risks, what are the benefits? Since we have no universal health identifier in the US, there is no simple way to uniquely identify a patient at all sites of care. The result is a fractured medical record scattered in inpatient, outpatient, laboratory, pharmacy, and emergency department sites. The implanted RFID devices enable patients to establish health care identities and become the stewards of their own data. The patient can assemble a reconciled medication list, a complete problem list, and a list of diagnostic study results, and then apply personal privacy preferences—for example, deleting information about mental health, HIV, or substance abuse. This patient-controlled record is available to treating clinicians in the case of emergency via the implanted device.

It is a personal choice whether or not to be fitted with an RFID device, but for some patients such a record has value. For example, such devices may be particularly helpful for a patient with Alzheimer disease who cannot give a history, a patient prone to syncope who may not be initially conscious during an emergency department visit, or a very active person who engages in extreme sports activities and could be noncommunicative due to injury.

I believe that in the near future, patients will own their medical records and be the stewards of their own health care data. Implantation of RFID devices is one tool, appropriate for some patients based on their personal analysis of risks and benefits, that can empower patients by serving as a source of identity and a link to a personal health record when the patient cannot otherwise communicate.

## Abbreviations

AMA: American Medical Association
FDA: Food and Drug Administration
RFID: radiofrequency identification
