# Correction: What Are the Benefits and Risks of Fitting Patients with Radiofrequency Identification Devices?

**DOI:** 10.1371/journal.pmed.0050036

**Published:** 2008-02-05

**Authors:** Mark Levine, Ben Adida, Kenneth Mandl, Isaac Kohane, John Halamka

Correction for:

Levine M, Adida B, Mandl K, Kohane I, Halamka J (2007) What are the benefits and risks of fitting patients with radiofrequency identification devices? PLoS Med 4(11): e322. doi:10.1371/journal.pmed.0040322

John Halamka declared that he had no competing interests. While he has no financial relationship with the VeriChip Corporation, he serves as an unpaid adviser to the corporation on privacy, security, and standards issues, as well as strategy and future applications of the VeriChip radiofrequency identification implantable microchip technology.

